# Sense of purpose in life and work-life tension: Perceptions of interference and enhancement

**DOI:** 10.1016/j.ahr.2023.100154

**Published:** 2023-07-22

**Authors:** Angelina R. Sutin, Martina Luchetti, Yannick Stephan, Antonio Terracciano

**Affiliations:** a Department of Behavioral Sciences and Social Medicine, Florida State University College of Medicine, 1115W. Call Street, Tallahassee, FL 32306, United States; b Euromov, University of Montpellier, Montpellier France; c Department of Geriatrics, Florida State University College of Medicine, Tallahassee, United States

**Keywords:** Purpose in life, Work-life tension, Interference, Enhancement, Cognitive aging

## Abstract

This research examines the relation between purpose in life and perceptions of work-life interference (work interferes with personal life and vice versa) and enhancement (work enhances personal life and vice versa) and whether these dimensions mediate purpose and cognition over 10 years. Employed participants from the Health and Retirement Study (*N* = 4,492) reported on their purpose in life and work-life interference and enhancement; a subset (*N* = 2,207) had cognition measured at baseline and again 10 years later. Purpose was associated with less work-life interference and greater work-life enhancement. Purpose was associated with maintenance of cognition, but the work-life dimensions were unrelated to cognition and thus did not account for the relation between purpose and maintenance of cognitive function. This research suggests that purpose in life is associated with greater integration of working and personal lives. Such integration can promote better aging-related outcomes, but not cognitive function.

## 1. Introduction

Purpose in life is a component of well-being [1] that is associated with aspects of employment: Individuals often derive purpose from their work [2], whereas leaving the workforce can diminish purpose [3]. There are aspects of employment prior to retirement that may be associated with purpose. There is often a need, for example, to integrate work and personal lives, an integration that can be positive or negative: work can both enhance and interfere with personal lives, and, likewise, personal lives can enhance and interfere with work [4]. Individuals higher in purpose tend to balance competing demands and cope better with stress [5] and such strategies may extend to how well working and personal lives are integrated. Further, given that more enhancement and less interference are associated with better health [6], these dimensions may be mechanisms between purpose and critical outcomes, such as cognition. Purpose is associated with better cognition [7], in part, but not completely, through better behavioral and clinical profiles [8]. Work-life tension may also operate in this pathway: Perceptions of integration between work and personal life may support better cognition, whereas greater tension may lead to stress or unhealthy behaviors harmful to cognition. If greater integration and less tension are associated with both purpose and cognition, they may be mechanisms between purpose and healthier cognition.

The present research examines purpose in life and work-life tension among employed participants and whether work-life tension is a mechanism between purpose and maintenance of cognition over 10 years. We expect purpose to be associated with greater enhancement and less interference across work and personal lives and that this balance will mediate purpose and cognition.

## 2. Method

### 2.1. Participants and procedures

Participants from the Health and Retirement Study (HRS) [9] reported on their purpose and work-life tension as part of the Leave Behind Questionnaire (LBQ) in 2006 or 2008. Participants were selected into the cross-sectional sample if they reported currently working and completed the LBQ. For the mediation analysis, participants were selected into the longitudinal sample if they also had cognitive data at baseline (2006/2008) and 10 years later (2016/2018). There were 4492 participants in the cross-sectional sample and 2707 participants in the longitudinal mediation sample. See supplementary material for attrition.

### 2.2. Measures

#### 2.2.1. Purpose in life

Purpose was assessed with seven items from the Ryff Measures of Psychological Well-being [1]. Items were rated from 1 (*strongly disagree*) to 6 (*strongly agree*), reverse scored when necessary, and the mean taken in the direction of greater purpose.

#### 2.2.2. Work-life tension

A 12-item measure of work-life tension [10] assessed how much work interfered with personal life, personal life interfered with work, work facilitated personal life and personal life facilitated work. Items were rated from 1 (*rarely*) to 4 (*most of the time*). The mean was taken in the direction of each dimension label.

#### 2.2.3. Cognition

Participants were administered the modified Telephone Interview for Cognitive Status (TICSm) [11]. The score is the sum of immediate and delayed recall of 10 words (20 points), serial 7 subtraction (5 points), and backward counting (2 points). Cognitive assessments were concurrent with baseline (2006/2008) and 10 years later (2016/2018).

#### 2.2.4. Covariates

Covariates were age in years, sex (0=male, 1=female), race (two dummy coded variables that compared Black participants [=1] and otherwise identified participants [=1] to white participant [=0]; i.e., all participants not self-identified as white were coded as 1; the dummy codes compared Black participants to white participants and otherwise identified participants to white participants), Hispanic ethnicity (0=no, 1=yes), education in years, and year of LBQ (0 = 2006, 1 = 2008).

### 2.3. Analytic strategy

We used linear regression to test purpose in life and the four work-life tension dimensions, controlling for covariates. Exploratory analyses examined whether these associations varied by age, sex, race, ethnicity, or education by testing an interaction between purpose and each factor. We then tested whether work-life tension mediated the prospective association between purpose at baseline and cognition 10 years later (defined as TICSm performance at the 2016/2018 follow-up). We used Model 4 from the PROCESS macro [12] to test the work-life dimensions as simultaneous mediators, controlling for covariates and baseline cognition. Significance was set to *p* < 0.01 because of sample size and number of tests.

## 3. Results

Descriptive statistics, bivariate correlations, and results of the linear regressions are in Supplemental Tables S1–S3. Purpose was associated with less work-life tension and greater integration: Participants higher in purpose perceived that their personal life did not interfere with their work (β=−0.22) and that their work did not interfere with their personal life (β=−0.21), and, further, that their work facilitated their personal life (β=0.31) and that their personal life facilitated their work (β=0.36). There was little evidence for moderation. The association between purpose and personal life facilitated work was apparent across age but stronger at relatively younger ages (β_purpose × age_=−0.04, *p* < 0.010). Education moderated purpose and work interfered with personal life (β_purpose × education_=0.04, *p* < 0.010) and personal life interfered with work (β_purpose × education_=0.07, *p* < 0.010) such that these associations were apparent across education but stronger at relatively lower education (purpose was more protective among participants with relatively lower education). The other interactions were not significant.

Results of the mediation analysis are in Table 1. Consistent with previous research in HRS [13], purpose was associated with maintaining cognition over follow-up. None of the work-life tension factors, however, was associated with cognition and thus did not mediate the association between purpose and maintaining cognition over 10 years.

## 4. Discussion

Purpose in life is associated with less work-life interference and greater work-life enhancement. This pattern is consistent with the literature on purpose and balancing competing demands [5] and extends it to a consequential context – work-life tension – that constitutes a significant part of life for many working adults. Surprisingly, work-life tension was unrelated to cognition and thus did not mediate purpose and maintenance of cognition over the 10-year follow-up.

Purpose in life is an aspect of psychological well-being associated consistently with better outcomes [7], including employment-related outcomes (e.g., income [14]). Purpose is reactive to employment, such that work life can be critical for how individuals assess their purpose [2] and it tends to react to transitions in employment, such as retirement [3]. Less research has addressed purpose and dimensions of working life, including perceptions of work-life tension. It is not uncommon for individuals to balance the demands of their work and personal lives [4] and such balance has implications for aging. For example, greater integration is associated with better health outcomes with age [6], whereas greater feelings of tension are harmful for health [15].

There are several reasons why purpose may be associated with more enhancement and less interference. First, purpose is associated with emotion regulation strategies that may support better work-life balance, including the use of reappraisal and planning strategies and fewer self-blame and catastrophizing strategies [16]. Individuals higher in purpose may thus reappraise stressors to be less conflictual and have better organizational skills to manage busy work and personal lives. When confronting stressors at work, a problem-focused approach makes it less likely that such stressors interfere with other life domains. Second, individuals higher in purpose feel they have agency [17], which may reduce potential conflicts between work and personal life. That is, such individuals may have more control over aspects of their work and personal lives that reduce conflict and promote enhancement. Finally, individuals higher in purpose derive satisfaction from their jobs [18] and relationships [19] and may find the two more complementary than conflictual.

There was little evidence for moderation by sociodemographic characteristics. Given that women tend to be less satisfied with their work-life balance than men [20], purpose might have been more relevant for managing this tension for women. There was no evidence of such an interaction. Purpose did seem to serve as a resource for fewer perceptions of interference for individuals with less education. This moderation is consistent with related work that shows purpose can be a psychological resource in more challenging economic environments [13].

There is a robust association between purpose in life and healthier cognitive aging and lower risk of dementia [7]. Healthier behavioral and clinical patterns account for some, but not all, of this association, which suggests other factors may be important mechanisms in this pathway [8]. Given that greater work-life integration can promote healthier outcomes [6], whereas work-life tension can be harmful [15], it was possible that these associations would extend to cognitive health. These dimensions, however, were unrelated to cognition at follow-up. As such, even though purpose was associated with healthier work-life integration and cognition, these perceptions did not contribute to maintenance of cognition over time.

This research had several strengths, including the large sample and 10 years of follow-up for cognition. There are also limitations. First, the sample was specific to the American employment context and may not generalize to other countries. Second, the sample did not include young adults who may have different demands when starting a career and family than middle aged and older adults who may have established working and family lives. Third, there may have been reverse causality, omitted variables bias, and selection bias due to attrition.

## 5. Conclusion

The present research found that among employed individuals, higher purpose in life was associated with less work-life interference and greater work-life enhancement. Further, although purpose was associated with cognitive function over 10 years, the work-life dimensions were unrelated to cognition and thus did not mediate purpose and maintenance of cognition over 10 years. From a practical perspective, purpose in life could be an intervention target to help support better balance between the demands of work and life. The results also indicate that balance is not a mechanism that explains why purpose supports cognitive function over time and thus future research should test other aspects of employment to identify potential work-related mechanisms that are promising targets to support cognitive health.

## Supplementary Material

1

## Figures and Tables

**Table 1:** Indirect Effects of Purpose in Life on Cognitive Function Through Work-Life Tension.

	Mediation Parameter							
	SES to Work-Life (path a)		Work-life to Cognitive Function (path b)		Indirect Effect (axb)		Total Effect (path c)	Direct Effect (path c’)
	b (SE)	p	b (SE)	p	b (SE)	p	b (SE)	b (SE)
Purpose in Life							0.08 (0.02)	0.07 (0.02)
Work interferes with personal life	−0.19 (0.02)	<0.001	0.00 (0.02)	0.834	0.00 (0.00)	0.835	*p* < 0.001	*p* < 0.001
Personal life interferes with work	−0.21 (0.02)	<0.001	0.02 (0.02)	0.393	0.00 (0.00)	0.396		
Work enhances personal life	0.31 (0.02)	<0.001	−0.01 (0.02)	0.763	0.00 (0.01)	0.763		
Personal life enhances work	0.36 (0.02)	<0.001	0.03 (0.02)	0.240	0.01 (0.01)	0.241		
