# Supplementary material for: Sense of purpose in life and work-life tension: Perceptions of interference and enhancement
Source: Aging Health Res. Author manuscript; Available in PMC 2023 Oct 18. (PMC10584036; doi:10.1016/j.ahr.2023.100154)
Supplement: 1 [file NIHMS1931537-supplement-1.docx]

Supplemental Material

A random half of participants in the Health and Retirement Study completed the Leave Behind Questionnaire (LBQ) in 2006; the other half completed it in 2008. These two samples were combined as baseline. Participants were selected for the cross-sectional analysis if they completed the LBQ and reported that they were currently working (*N*=4,492). These participants had complete data on the sociodemographic covariates (age, sex, race, ethnicity, education. Of these participants, *n*=2,707 had cognitive data available at baseline and completed the same cognitive assessment again 10 years later. This sample was used for the longitudinal mediation analysis. Participants without follow-up data (*n*=1,785) were older (*d*=0.27, *p*<0.010), more likely to be male (χ^2^=12.52, *p*<0.010), and had lower baseline cognitive function (*d*=0.23, *p*<0.010). There was no difference in race (χ^2^=2.05, *ns*), education (*d*=0.05, *ns*), or purpose in life (*d*=0.02, *ns*). The associations between purpose and the dimensions of work life balance were similar when the full cross-sectional sample was analyzed or the subsample that also had cognition available at baseline and 10 years later (Supplemental Table S3 and Table 1).

Supplemental Table S1

*Mean (standard deviation) or Percentage (n) for All Study Variables*

| Variable | Mean (SD) or % (n) |
| --- | --- |
| Age (years) | 60.76 (9.91) |
| Sex (female) | 55.9 (2510) |
| Race (Black) | 11.9 (534) |
| Race (Otherwise identified) | 5.8 (259) |
| Hispanic Ethnicity (yes) | 7.7 (346) |
| Education (years) | 13.52 (2.71) |
| Year (2008) | 44.6 (2005) |
| Purpose in life | 4.80 (0.87) |
| Work-Life Tension |  |
| Work interferes with personal life | 1.54 (0.57) |
| Personal life interferes with work | 1.17 (0.34) |
| Work facilitates personal life | 2.82 (0.88) |
| Personal life facilitates work | 3.21 (0.79) |
| Cognition (*n*=2,707) |  |
| Baseline cognition | 17.29 (3.51) |
| 10-year follow-up | 16.08 (4.31) |

*Note*. *N*=4,492.

Supplemental Table S2

*Correlations Among All Study Variables*

|  | 1. | 2. | 3. | 4. | 5. | 6. | 7. | 8. | 9. | 10. |
| --- | --- | --- | --- | --- | --- | --- | --- | --- | --- | --- |
| 1. Age (years) | -- |  |  |  |  |  |  |  |  |  |
| 2. Sex (female) | -0.15* | -- |  |  |  |  |  |  |  |  |
| 3. Race (Black) | 0.02 | 0.07* | -- |  |  |  |  |  |  |  |
| 4. Race (Otherwise identified) | -0.12* | 0.00 | -0.09* | -- |  |  |  |  |  |  |
| 5. Hispanic ethnicity (yes) | -0.10* | 0.01 | -0.09* | 0.40* | -- |  |  |  |  |  |
| 6. Education (years) | -0.07* | -0.05* | -0.09* | -0.06* | -0.28* | -- |  |  |  |  |
| 7. Year of LBQ (2008) | 0.10* | 0.02 | 0.03 | 0.00 | 0.02 | -0.02 | -- |  |  |  |
| 8. Purpose in life | 0.02 | 0.02 | 0.06* | -0.02 | -0.03 | 0.16* | 0.06* | -- |  |  |
| 9. Work interferes with personal life | -0.08* | 0.03 | 0.00 | 0.06* | 0.04* | -0.06* | 0.01 | -0.21* | -- |  |
| 10. Personal life interferes with work | -0.16* | 0.00 | -0.07* | 0.02 | -0.01 | 0.02 | 0.00 | -0.22* | 0.40* | -- |
| 11. Work facilitates personal life | 0.20* | -0.02 | 0.00 | -0.01 | 0.02 | 0.03 | 0.04* | 0.31* | -0.24* | -0.53* |
| 12. Personal life facilitates work | 0.12* | -0.04* | -0.06* | -0.02 | 0.00 | 0.05* | 0.02 | .35* | -.35* | -.35* |
| 13. Baseline cognition (n=2,707) | -0.14* | .007* | -0.24* | -0.11* | -0.15* | 0.37* | -0.08* | .11* | -.03 | .04 |
| 14. Follow-up cognition (n=2,707) | -0.25* | 0.12* | -0.15* | -0.06* | -0.07* | 0.30* | 0.04 | .14* | -.01 | .05* |

*Note*. *N*=4,492.

**p*<0.010.

*Table S1 Continued*

|  | 11. | 12. | 13. | 14. |
| --- | --- | --- | --- | --- |
| 11. Work facilitates personal life | -- |  |  |  |
| 12. Personal life facilitates work | 0.65* | -- |  |  |
| 13. Baseline cognition (n=2,707) | 0.00 | 0.04 | -- |  |
| 14. Follow-up cognition (n=2,707) | -0.01 | 0.04 | 0.48* | -- |

*Note*. *N*=4,492.

**p*<0.010.

Supplemental Table S3

*Association between Purpose in Life and Dimensions of Work-Life Tenison*

| Predictors |  | Work interferes with personal life | Personal life interferes with work | Work facilitates personal life | Personal life facilitates work |
| --- | --- | --- | --- | --- | --- |
| Age (years) |  | -0.07* | -0.16* | 0.20* | 0.12* |
| Sex (female) |  | 0.02 | -0.01 | 0.01 | -0.02 |
| Race (Black) |  | 0.01 | -0.06* | -0.02 | -0.08* |
| Race (otherwise identified) |  | 0.04 | 0.01 | 0.00 | -0.02 |
| Hispanic ethnicity (yes) |  | 0.00 | -0.02 | 0.04* | 0.02 |
| Education (years) |  | -0.03 | 0.04 | 0.00 | 0.00 |
| Year (2008) |  | 0.03 | 0.04 | 0.01 | -0.01 |
| Purpose in life |  | -0.21* | -0.22* | 0.31* | 0.36* |

Note. N=4,492. Coefficients are standardized beta coefficients from linear regression.

*p<0.010.
